# Extreme Temperatures Reduce Copepod Performance and Change the Relative Abundance of Internal Microbiota

**DOI:** 10.1002/ece3.70408

**Published:** 2024-10-11

**Authors:** Quyen D. H. Vu, Linh P. Pham, Oanh T. Truong, Sang Q. Tran, Canh V. Bui, Minh‐Hoang Le, Binh T. Dang, Khuong V. Dinh

**Affiliations:** ^1^ Institute for Biotechnology and Environment, Nha Trang University Nha Trang City Vietnam; ^2^ Cam Ranh Centre for Tropical Marine Research and Aquaculture Institute of Aquaculture, Nha Trang University Nha Trang City Vietnam; ^3^ Section for Aquatic Biology and Toxicology, Department of Biosciences University of Oslo Oslo Norway

**Keywords:** climate change, copepods, marine heatwaves, temperature, tropical marine ecosystems

## Abstract

Copepods are one of the most abundant invertebrate groups in the seas and oceans and are a significant food source for marine animals. Copepods are also particularly sensitive to elevated temperatures. However, it is relatively unknown how the internal microbiome influences copepod susceptibility to warming. We addressed this fundamental knowledge gap by assessing key life history traits (survival, development, and reproduction) and changes in the internal microbiome in the tropical calanoid copepod *Acartia* sp. in response to warming (26°C, 30°C, and 34°C). Copepod microbiomes were analyzed using high throughput DNA sequencing of V1–V9 of 16S rRNA hypervariable regions. Copepod performance was better at 30°C than at 26°C, as indicated by faster development, a higher growth rate, and fecundity. However, these parameters strongly decreased at 34°C. We recorded 1,262,987 amplicon sequence reads, corresponding to 392 total operational taxonomic units (OTUs) at 97% similarity. Warming did not affect OTU numbers and the biodiversity indices, but it substantially changed the relative abundance of three major phyla: Proteobacteria, Actinobacteria, and Bacteroidota. The thermophilic and opportunistic Proteobacteria and Bacteroidota increased under extreme temperatures (34°C) while Actinobacteria abundance was strongly reduced. Changes in the relative abundance of these bacteria might be related to reduced copepod growth, survival, and reproduction under extreme temperatures. Profiling the functional role of all internal bacterial groups in response to the temperature change will fundamentally advance our mechanistic understanding of the performance of tropical copepods and, more generally, marine invertebrates to a warming climate.

## Graphical abstract

Marine heatwaves (MHWs) emerge as a severe stressor in marine ecosystems, yet it is relatively unknown the role of the internal microbiome in shaping copepod susceptibility to MHWs. Our study showed that *Acartia* copepods decreased survival, growth, and reproduction at 34°C, a typical MHW temperature. The thermophilic and opportunistic Proteobacteria and Bacteroidota increased under extreme temperatures while Actinobacteria abundance was strongly reduced; all might be related to reduced copepod growth, survival, and reproduction.

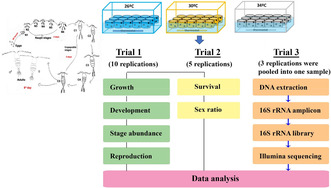

## 1 Introduction

Climate change is one of the greatest threats to marine organisms worldwide (Brander 2007; Merino et al. 2012; Barange et al. 2014; Handisyde, Telfer, and Ross 2017). This is especially true in tropical regions where many species already live close to their upper physiological tolerance (Dinh et al. 2022; Tewksbury, Huey, and Deutsch 2008). Global warming increases aquatic animals’ metabolism while reducing dissolved oxygen in the water (Brander 2007), thus enhancing negative effects on marine taxa (Sampaio et al. 2021). Among others, copepods are key secondary producers, contributing to 80% of mesozooplankton biomass in the seas and oceans (Moriarty and O'Brien 2013; Blaxter et al. 1998). However, under climate change, the abundance of tropical copepods is rapidly decreasing, up to 50% by 2100 (Ibarbalz et al. 2019), but we poorly know how these tropical copepods cope with thermal stress.

Previous studies have shown that the performance of both temperate and tropical copepods is reduced at temperatures > 30°C (Doan et al. 2018, 2019; Sasaki et al. 2019; Nguyen et al. 2020; Truong et al. 2020, 2022) which is in agreement with the low abundance of copepods in coastal ponds despite a high food availability (Grønning et al. 2019). However, climate change research on marine systems has mostly focused on exploring the physiological and behavioral mechanisms underlying the responses of copepods to warming. Heat stress often causes copepods to engage in multiple physiological responses, which can result in tradeoffs between increased energy demand for coping with thermal stress *versus* growth and reproduction; meanwhile, the energy intake may be reduced because of feeding the inefficiency. For example, the tropical copepod *Pseudodiaptomus annandalei* showed a temperature coefficient (*Q*_10_) value of 4.44 for the weight‐specific oxygen consumption at 32°C–36°C, which was two times higher than a *Q*_10_ value of 2.01 at 26°C–32°C, suggesting a much higher increase of metabolic expenses under extreme temperature (Lehette et al. 2016). The upregulation of the heat shock proteins in *P*. *annandalei*, which is critical for coping with thermal stress, showed the highest level at 32°C and decreased at higher temperatures (Low et al. 2018). Furthermore, the feeding was also reduced at temperatures beyond 32°C, which could lower the energy and resources available to meet the increased metabolic rate in extreme temperatures (Doan et al. 2018, 2019). Copepods actively avoid extreme temperatures even if they have to cope with low dissolved oxygen in the water as one of the typical behavioral responses to warming, including the marine heatwaves (MHWs) (Dinh, Cuevas‐Sanchez, et al. 2020; Dinh, Dinh, et al. 2020).

Recent technological breakthroughs such as high‐throughput sequencing technology have opened up enormous possibilities for further investigation of the influence of internal microbial communities on host responses to climate change from individual host species (Alberdi et al. 2016; Hector et al. 2022; Marsh, Bearhop, and Harrison 2024; Qin et al. 2010) to the food web structure and ecological networks (Bohan et al. 2017). While internal microbiomes may include microbiomes from a diversity of animal compartments or organs, these bacteria are mostly associated with the digestive or respiration systems (Woodhams et al. 2020). The internal microbiota can affect digestion (Leigh, Papastamatiou, and German 2021; Liao et al. 2023), energy metabolism (Carey, Walters, and Knight 2013), immune response (Ruiz‐Rodríguez et al. 2009), and toxin resistance (Gorokhova et al. 2021) of the host. Consequently, changes in the internal microbiome can lead to physiological disturbances in the host. For example, climate warming of 2°C–3°C caused a 34% loss of lizard (*Zootoca vivipara*) population gut microbiota diversity, which correlated negatively with their survival (Bestion et al. 2017). It has been shown that an increased *Vibrio* bacteria in the soft tissues of corals and sponges have been shown to cause diseases in mass mortality under MHWs (Vezzulli et al. 2010; Dinçtürk et al. 2023). Transplantation of internal microbes from heat‐acclimated animals (e.g., sea anemones *Nematostella vectensis*) increased the thermal tolerance of non‐acclimated individuals (Baldassarre et al. 2022), while transplantation of the gut microbiomes from cold‐adapted individuals (e.g., in *Drosophila melanogaster*) reduced the thermal tolerance of the recipients (Moghadam et al. 2018). Given the internal microbiome can have strong influences on various physiological processes of the host, therefore exploring the role of internal microbiomes of copepods in relation to elevated temperatures caused by climate change can provide additional mechanisms on how copepods may cope with thermal stress. However, this topic has rarely been investigated, especially for the tropical copepods.

Here, we experimentally tested how warming affects key life history traits of a tropical copepod *Acartia* sp., such as development, growth (body size), survival, and reproduction. We took the first step in exploring how changes in copepod internal microbiome diversity change at different warming levels. A previous study has shown an increased relative abundance of Proteobacteria in the gut microbiome of copepod (*Tigriopus kingsejongensis*), an Antarctic, cold‐adapted species, in relation to the elevated temperatures from 2°C to 15°C (Oh et al. 2022). Our study will provide the first understanding of the internal microbiota dynamics in relation to the responses of tropical copepods to extreme warming from 26°C to 34°C in the tropical seas and oceans.

## 2 Materials and Methods

### 2.1 *Acartia* sp. Sampling and the Experimental Setup

We collected the copepod *Acartia* sp. in a coastal pond (11.8247 N, 109.1255 E) in Cam Ranh, Khanh Hoa province, Vietnam, using a plankton net (mesh size = 200 μm). The morphology of this species is similar to *Acartia tropica*. However, the 28S rRNA and COI mtDNA sequences of this species showed the highest similarity (92.3%) with *Acartia southwelli*, and therefore could be potentially a new *Acartia* species (Vu et al. in prep).

At the sampling site, temperature and salinity in the pond were 28°C–29°C and 25 PSU, respectively. Copepods were transferred to the Wet Lab at Nha Trang University. In the laboratory, copepods were acclimated to room temperature (around 28°C), experimental salinity of 25 PSU, and a photoperiod of 12 h light:12 h dark for 10 days using the natural light cycle at the study location. They were fed two times a day with *Isochrysis galbana* at a density of about 40,000 cells/L. Culturing protocols were adapted from Doan et al. (2018).

After the acclimation period, healthy and active copepods at the adult stage were transferred into new tanks (*V* = 8 L) for egg production using 100 μm mesh sieves. After 48 h, eggs were collected by using 25 μm mesh sieves.

**FIGURE 1:**
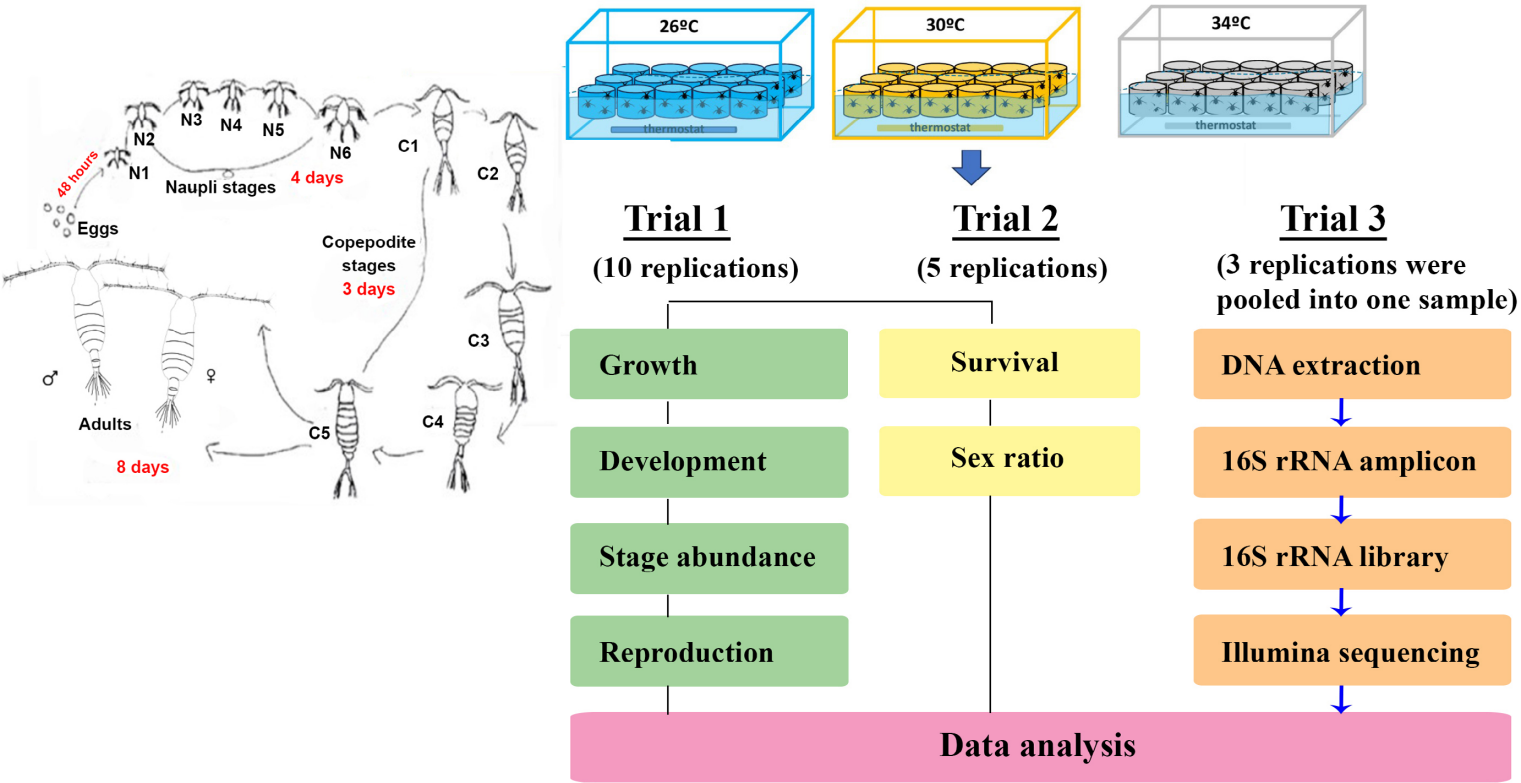
Schematic of experimental design. On the left is the life cycle of *Acartia* sp. from eggs to adult males and females: C1–C5, copepodite 1—copepodite 5; F, adult females; M, adult males; N1–N6, nauplii 1—nauplii 6. On the top‐right are the experimental temperatures of 26°C, 30°C, and 34°C with experimental vials inside the water baths. On the bottom‐right are three experimental trials with the number of replications per temperature and measurements in each of the trials.

Eggs were incubated at room temperature for 24 h. Newly hatched nauplii were then gathered on the surface with a flashlight to separate them from unhatched eggs and transferred to a new tank. From these tanks, nauplii were selected and distributed to experimental vials (*V* = 800 mL), each containing 400 individuals to start three trials: (1) the life history and reproduction trials (10 replications per temperature, or 30 experimental vials), (2) the survival, and sex ratio (5 replications per temperature, or 15 experimental vials), and (3) 16S rRNA amplicon sequencing (3 replications per temperature, or 9 experimental vials). The schematic of the experimental design is shown in Figure 1.

Experiments were conducted to investigate temperature effects on copepod performance. The temperature settings of 26°C, 30°C, and 34°C cover normal and extreme thermal variations in the study location (Doan et al. 2018, 2019). Experimental cups were placed in water baths and continuously monitored to achieve stable temperature for each treatment.

The temperature effect on the survival rate, developmental duration, growth, stage abundance and sex ratio was investigated for eight consecutive days, equivalent to the development time from the first nauplii to the adult stage of this species.

### 2.2 Trial 1: Growth, Development, Stage Abundance, and Reproduction

For measuring individual growth, developmental duration, and stage abundance, we took 10 subsamples (each 50 mL) from 10 replicates per temperature every 24 h until the population reached 100% maturity (day 8). Samples were passed through a 75 μm filter, and the content was poured on a petri dish and fixed by formaldehyde (4%) (Nguyen et al. 2020). Developmental stages were identified based on Marcus and Wilcox (2007). The body size was measured based on the body length of six nauplii stages (N1–N6) or prosome length of five copepodite stages (C1–C5) and adult males (M) and females (F) using a stereo microscope (Olympus SZ61). Developmental duration was determined by the time between nauplii 1% and 100% adults. The abundance of each stage was determined by counting the number of individuals at each stage, and the population structure was determined by the distribution (proportion) of each stage in the population.

The temperature effect on egg production and hatching success was investigated using copepod adults on day 8 of the above trial. For egg production, copepod adults from each temperature treatment were transferred in new 800 mL cups. A pair of one male and one female was collected from each of eight experimental cups (eight replications) per temperature to follow the egg reproduction. Each cup contained a filter net allowing only eggs to pass through but not adult copepods. Each pair of adults were transferred to a new cup every 48 h. Cups were placed in the same water bath that copepod adults were collected from during the experimental period. Eggs that remained in the cup were collected and counted. The process continued until no eggs were released (day 24).

To investigate the temperature effect on hatching rate, females with eggs on day 8 from each temperature were separated to collect eggs (after 48 h). After that, the number of eggs was distributed randomly into 12‐well plates. Plates (two plates for each temperature) were incubated at the same temperatures that females were collected from. At each temperature, newly hatched nauplii and eggs in each well were counted at 24 h in one plate and at 48 h in another plate. The hatching rate at each time point was calculated as the number of nauplii divided by the total number of nauplii and unhatched eggs.

### 2.3 Trial 2: Survival Rate and the Sex Ratio

Total copepods (the final copepodite stage—C5 and adults) in each of the five replicates were collected on day 8 to determine the survival rate and sex ratio.

$$\text{Survival rate}=\frac{100\times \text{total number of}\;C5\;\text{and adults}}{\text{Total number of nauplii stocked initially in each replicate}}$$

$$\text{The female proportion}=\frac{100\times \text{total number of}\;C5\;\text{and adult females}\;\mathrm{at}\;\mathrm{day}\;8}{\text{Total number of}\;\mathrm{all}\;C5\;\text{and adults}\;\mathrm{at}\;\mathrm{day}\;8}$$

### 2.4 Trial 3: The 16S rRNA Amplicon Sequencing and Data Processing

#### 2.4.1 DNA Extraction and Library Preparations

Nauplii, copepodite, and adult samples (nine samples per stage and 50 individuals per sample) were collected from three experimental temperatures (26°C, 30°C, and 34°C, three replicates per temperature), and stored in 1.5 mL Eppendorf tubes filled with 60% ethanol. Copepod samples were then transferred to a 20 μm filtered paper (Whatman, Merck) to partly remove the ethanol and remaining seawater. Filter papers were immediately placed in 1.5 mL Eppendorf tubes containing commercial bleach at a dilution of 2.5%. Previous studies have shown that the treatment with 2.5% commercial bleach could remove most of the extracellular DNA without affecting the detectability of the target internal DNA in the copepods (Greenstone et al. 2012). After 3 mins, copepod specimens on papers with bleach were transferred to Wizard SV Minicolumn Assembly (Promega, USA), then washed three times with distilled water. At this time, the chitin cover of copepods was softened, and copepodites and adult males and females were rinsed three times in nuclease‐free water. Afterward, we added nucleic lysis solution (200 μL), EDTA (50 μL), Proteinase K (20 μL), and RNase (5 μL), all from the kit Wizard genomic DNA purification (Promega).

Genomic DNA was isolated from the filter paper samples using Wizard SV Genomic DNA Purification System Kit (Promega) with two steps of modification: double the volume of the digestion solution and reduce the final elution volume to 100 μL. To assess DNA quality post‐extraction, DNA samples underwent electrophoresis on a 1.5% agarose gel and were quantified using the Qubit 2.0 Fluorometer (ThermoFisher Scientific, America). For Illumina sequencing, DNA concentration must be at least 10 ng/μL. DNA concentration and purity were measured at wavelength A260/280. DNA with a ratio ranging from 1.8 to 2.4 is considered pure. If the ratio is below 1.8, the sample is mixed with many impurities, mainly membrane proteins and the cover of copepods; if the ratio is above 2.4, membrane proteins remain in the sample. We excluded one sample (copepodites at 30°C) whose A260/280 ratio fell below 1.8 from the sequencing. Due to the low DNA obtained from each sample (< 10 ng/μL), we pooled three samples from a treatment (except copepodites at 30°C, we pooled only two samples) for 16S rRNA amplicon sequencing. We performed the amplification using primers for amplifying 16S rRNA V1‐V9 hypervariable regions as presented in Abellan‐Schneyder et al. (2021) and established the 16S rRNA libraries with V1–V9 hypervariable regions. We provided KTest Science Company, Ho Chi Minh City, Vietnam, with amplicons for sequencing using the Illumina MiSeq platform (2 × 150‐bp paired ends).

### 2.5 Microbiota Sequencing

The forward and reverse raw reads were trimmed with the barcode and low‐quality bases (Q‐score = 30) using Trimmomatic v0.33 (Bolger, Lohse, and Usadel 2014). The complete denoised sequences were assembled from paired‐end reads using the “mergePairs” function in DADA v2 (Callahan et al. 2016). Chimeric sequences (artifacts formed by incorrectly joined sequences) were detected and then removed using the “removeBimeraDenovo” function in DADA v2 to obtain high‐quality sequences. Operational taxonomic units (OTUs) at seven taxonomic levels with a variation of similarity (80%–97%) were identified from clustered sequences using the Blastn function in the QIIME2 pipeline (Bolyen et al. 2019). The relative abundance (%) of OTUs was calculated based on the number of clusters/total of obtained high‐quality sequences. QIIME2 taxa barplot's in two levels (Phylum and Genus) were applied to visualize the taxonomic composition between experimental samples based on 30 highly abundant taxa. Rarefaction curves were generated for visual comparison of observed OTUs/features at different sequencing depths (Figure S1 and Data S1). Alpha diversity indices, including Chao1, Shannon, and Simpson Diversity, were derived from the QIIME2 (http://qiime.org/) command α rarefaction to see how individual sample's diversity varies across strata. We calculated the distance matrix between OTUs in each sample as input data to perform a principal coordinate analysis (PCoA analysis) based on comparing the Bray–Curtis dissimilarity distance and Jaccard distance (Data S2).

### 2.6 Data Analyses

All statistical analysis and plotting were performed in R Studio software (v.1.1.463, R Core Team 2019). To investigate the temperature effects on developmental duration and relative abundance of stages in a population, we applied an ordinal logistic regression model (OLR, *ordinal* package, Christensen 2022). The ordinal dependent variable is a stage, and two independent factors are Temperature and Time (days). From the OLR, the abundance probability predicted for different stages in time (days) at each temperature was estimated. The median development time for each stage at different temperatures were calculated (*rcompanion*, Mangiafico 2024) and compared using Mood's median tests (*multcompView*, Graves, Piepho, and Dorai‐Raj 2024).

To determine the effects of temperature and stages on growth, we applied a linear regression model (LM) on ranked‐transformed data (*stats* package, R Core Team 2019). The temperature effects on survival, sex ratio, and hatching rate were determined by applying generalized linear regression models (GLM, *stats* package R Core Team 2019). To determine temperature and time effects on fecundity (replication is considered as a random factor), we applied a generalized linear mixed model via Panelized Quasi Likelihood (GLMMPQL, *MASS* package, Venables and Ripley 2002). Conditions for linear model assumptions (i.e., normality distribution and equal variance) were checked visually via diagnostic plots (Q–Q graphs, residuals against fitted values graphs). Pairwise comparisons were performed with the Tukey HSD post hoc test (*emmeans* package, Russell 2019). Significance was obtained when the *p* < 0.05.

To determine how OTUs and alpha biodiversity indices varied as a function of temperature or developmental stage, we used permutational multivariate analysis of variance (PERMANOVA) using QIIME2 (Bolyen et al. 2019). To assess species diversity at the genus level, we used the *tidyverse* package (Wickham et al. 2019) to analyze taxonomy data and species abundance greater than zero, calculated diversity indices Chao1 using the formula in the fossil package v0.4.0 (Vavrek 2011), and Shannon and Simpson using the vegan package v2.6‐2 (Oksanen et al. 2022), combined these indices into a data frame.

Figures are plotted using packages *Rmisc* (Hope 2013), *ggplot2* (Wickham 2016), and *ggpubr* (Kassambara 2019). A heatmap was generated using the package *pheatmap* (Kolde 2019) to visualize the relative abundance of the 14 most abundant phyla and 30 most abundant genera, in copepod samples in relation to temperatures and developmental stages.

## 3 Results

Overall, *Acartia* sp. showed faster development, a higher growth rate, and reproduction at 30°C than at 26°C, but all of these parameters and survival strongly decreased at 34°C. The internal microbiota biodiversity showed high dissimilarity between nauplii (N1–N6) with copepodites (C1–C5), and adults. Warming showed an insignificant effect on OTU numbers and alpha biodiversity indices but changed the relative abundance of bacterial groups among temperatures and life stages.

### 3.1 Life History Traits

**FIGURE 2:**
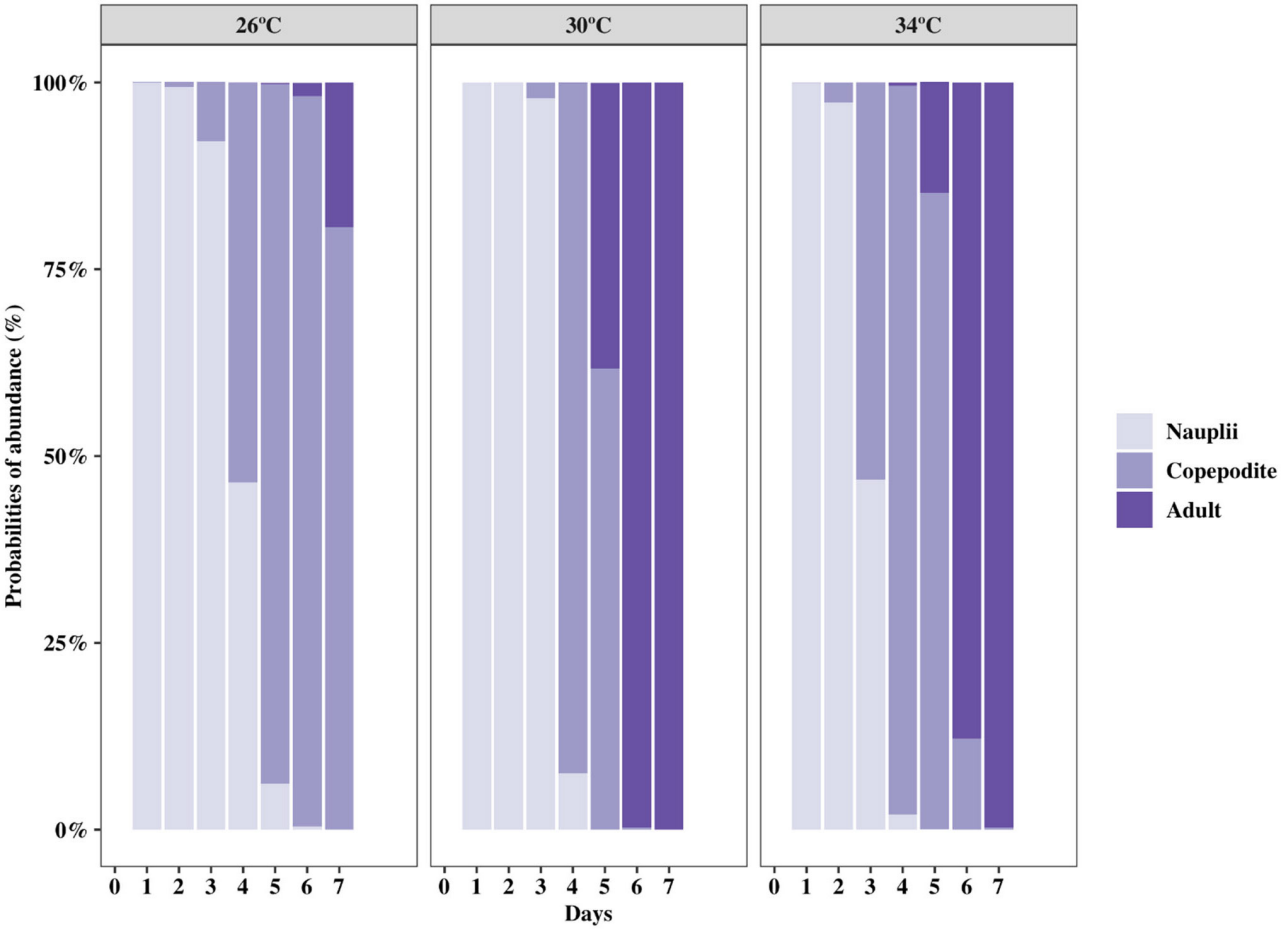
Relative abundance probability of the copepod *Acartia* sp. at different stages at 26°C, 30°C, and 34°C (panels) obtained from CLM (pseudo *R* ^2^ > 0.7). X‐axis shows the duration of development (Days); Y‐axis shows relative abundance (%). Each stacked bar represents the total number of individuals (100%) in the population each day.

Development time was longer at 26°C than at 30°C (*p* < 0.001) and 34°C (*p* < 0.001), while it was not significantly different between the latter two temperatures (Figure 2). The median development time of naupliar stages was 2 days at 30°C and 34°C, while it was 1 day longer at 26°C (Table 1). Copepodites dominated from day 3 (53%) at 34°C and day 4 (92%) at 30°C and lasted until day 5 in both temperatures. For 26°C, copepodites dominated from day 4 (54%) to day 6 (98%). All copepodites molted into adults on day 6 at 30°C and day 7 at 34°C, while at 26°C, only 25% of copepodites had molted into adults on day 7 (Figure 2 and Table 1).

**TABLE 1:** Median time in days of development duration for each stage at different temperatures.

Temperature °C	Stage	Day	*p* (pairwise Mood's median test)		
			26°C	30°C	34°C
26	Nauplii	3^a^		< 0.001	0.047
30	Nauplii	2^b^	< 0.001		0.117
34	Nauplii	2^b^	0.047	0.117	
26	Copepodite	6^a^		< 0.001	< 0.001
30	Copepodite	4^b^	< 0.001		0.695
34	Copepodite	4^b^	< 0.001	0.695	
26	Adult	8^a^		< 0.001	< 0.001
30	Adult	6^b^	< 0.001		< 0.001
34	Adult	7^c^	< 0.001	< 0.001	

*Note:* Different letters (“a,” “b,” and “c”) in the same column for each stage indicate significant differences among temperatures using pairwise Mood's median tests, *multcompView* package. Exact *p*‐values for pairwise comparisons are presented in columns 26°C, 30°C, and 34°C.

**FIGURE 3:**
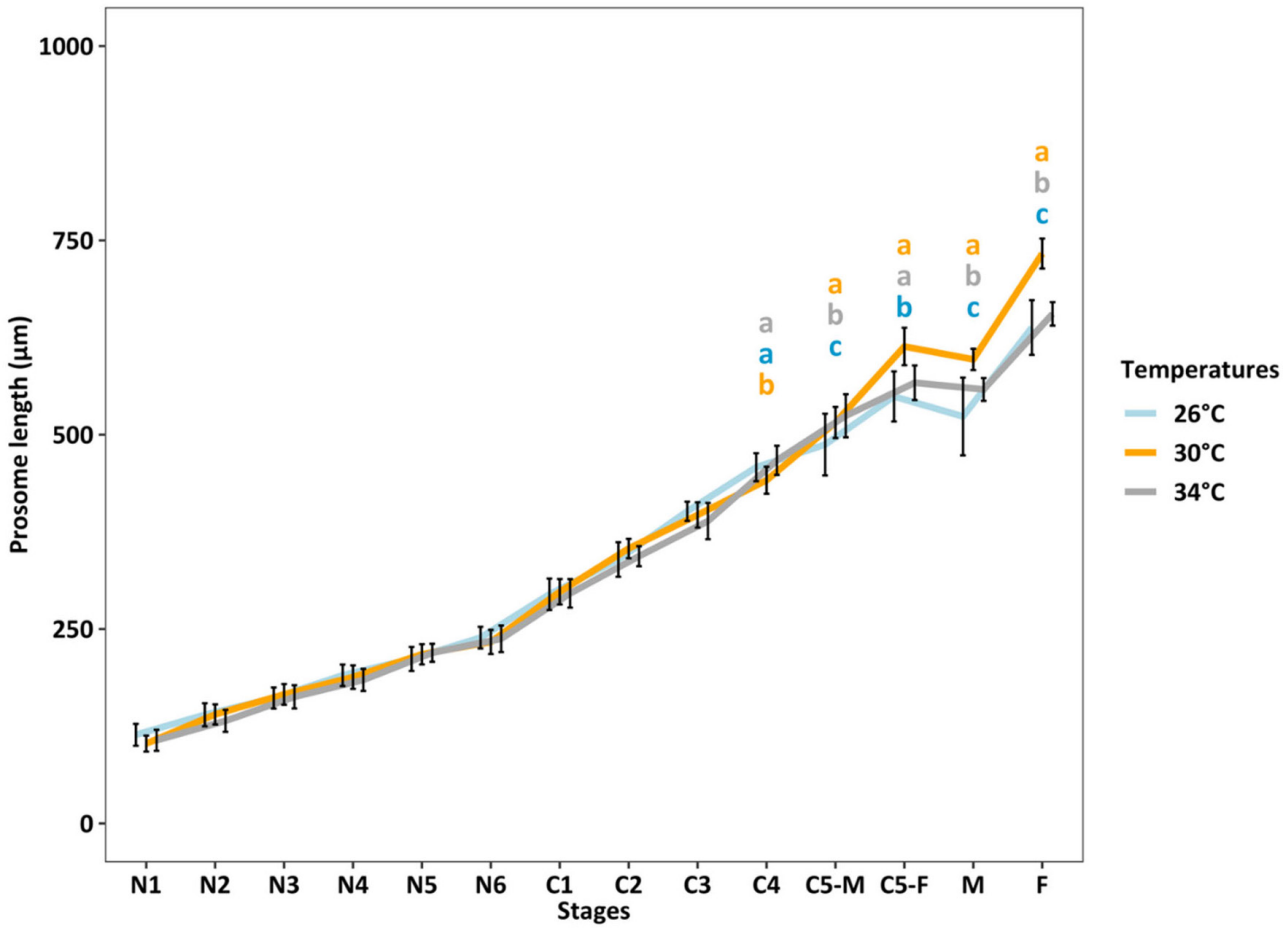
Copepod size (μm) of *Acartia* sp. at 26°C (blue), 30°C (orange), and 34°C (gray) (LM model for ranked transformed data, *R* ^2^ = 0.98, *p* < 0.001). Annotates: C1‐C4, copepodite 1—copepodite 4; C5‐M and C5‐F, copepodite 5 male and female; F, adult females; M, adult males; N1–N6, nauplii 1—nauplii 6. The number of specimens varied from 9 to 55 individuals per stage per temperature. Different letters “a,” “b,” and “c” indicate significant differences in sizes of copepod stages among temperatures (Tukey HSD posthoc test, *p* < 0.001); at the same stage, the order of letters in 1 column from top to bottom indicates treatment from 26°C, 30°C, and 34°C, respectively. The line chart and error bar present data as mean ± SE. Details on pairwise comparisons Emmeans are provided in Data S3.

Copepod length increased throughout their lifetime from N1 to adults (*p* < 0.001). From N1 to C3, no temperature effect on copepod size was observed. From C5 to adults, copepods were largest at 30°C (*p* < 0.001) and smallest at 26°C (*p* < 0.001), and the difference in the copepod length was more pronounced in adult females (Figure 3).

**FIGURE 4:**
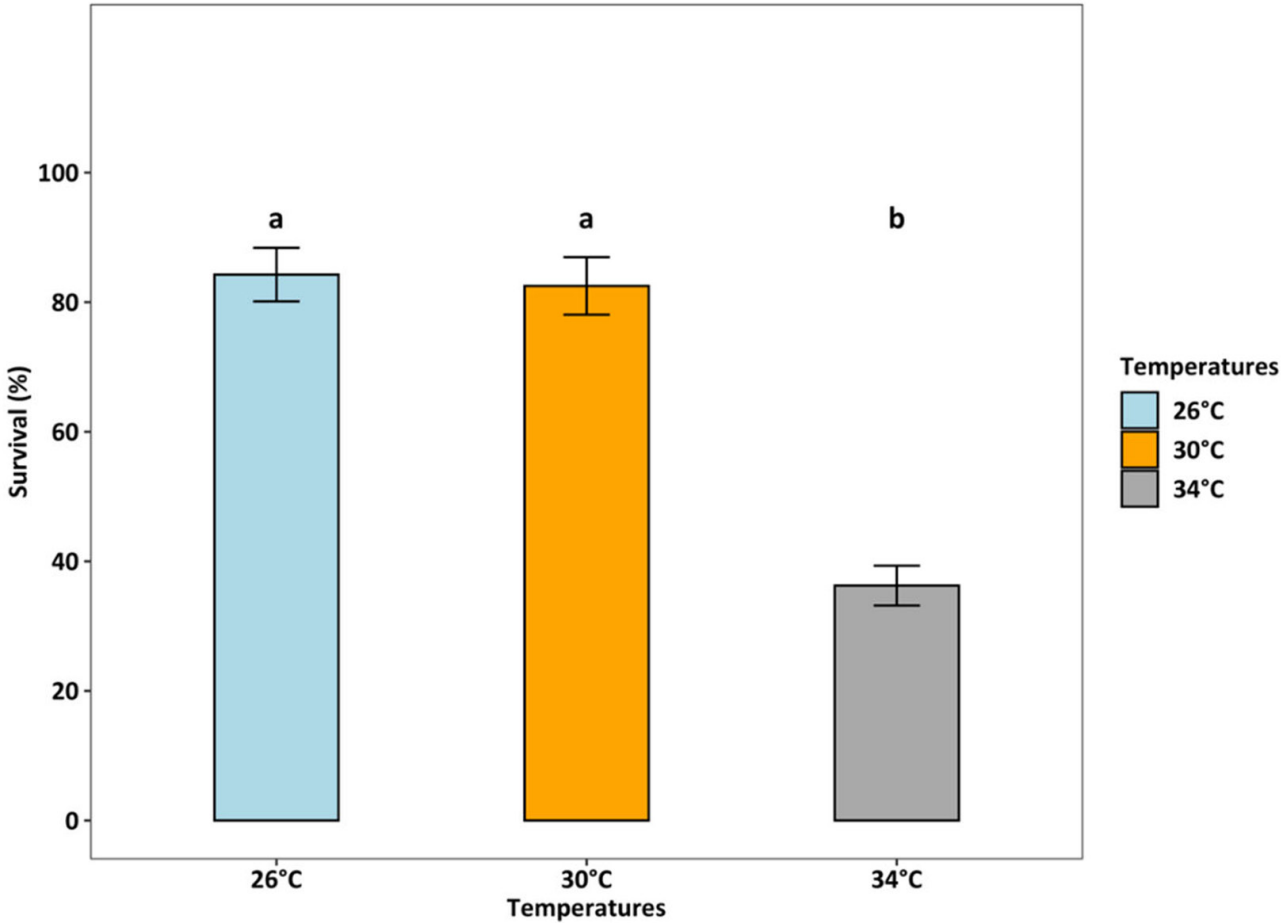
The percentage of surviving *Acartia* sp. from nauplii to adults in response to experimental temperatures (GLM model for quasibinomial distribution, *R* ^2^ = 0.88). Different letters “a” and “b,” indicate significant differences among treatments (Tukey HSD posthoc test, *p* < 0.01). Bars and error bars present data as Mean ± SE. Details on pairwise comparisons are provided in Data S3.

**FIGURE 5:**
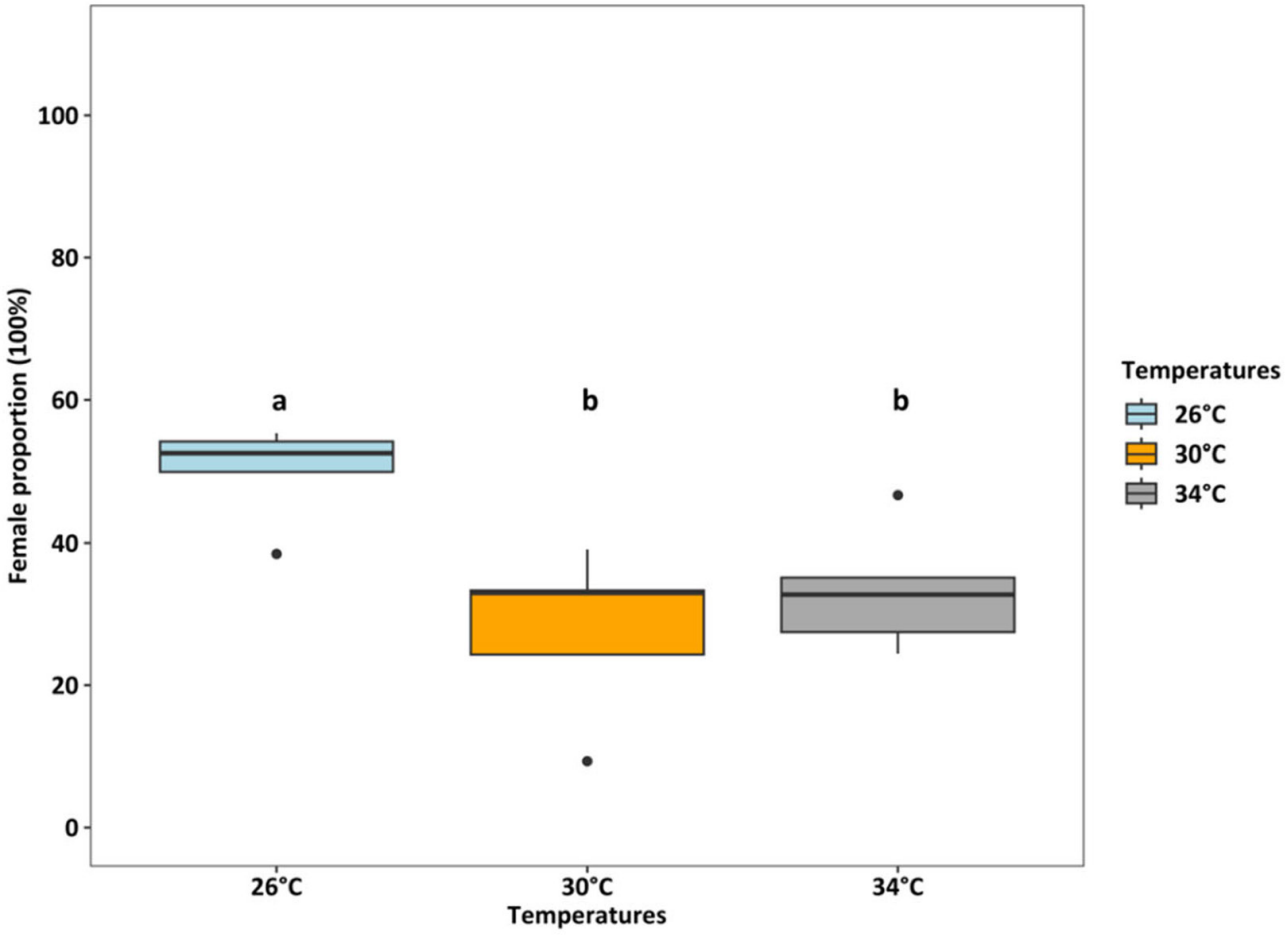
The proportion of *Acartia* sp. females at different temperatures (GLM model for quasibinomial distribution, *R* ^2^ = 0.49). Each box presented a median (fine line in the middle of the box), the 1st and 3rd quantiles (upper tale and lower tale), and outliers (dots). Different letters “a” and “b,” indicate significant differences among treatments (Tukey HSD posthoc test, *p* < 0.01). Details on pairwise comparisons are provided in Data S3.

**FIGURE 6:**
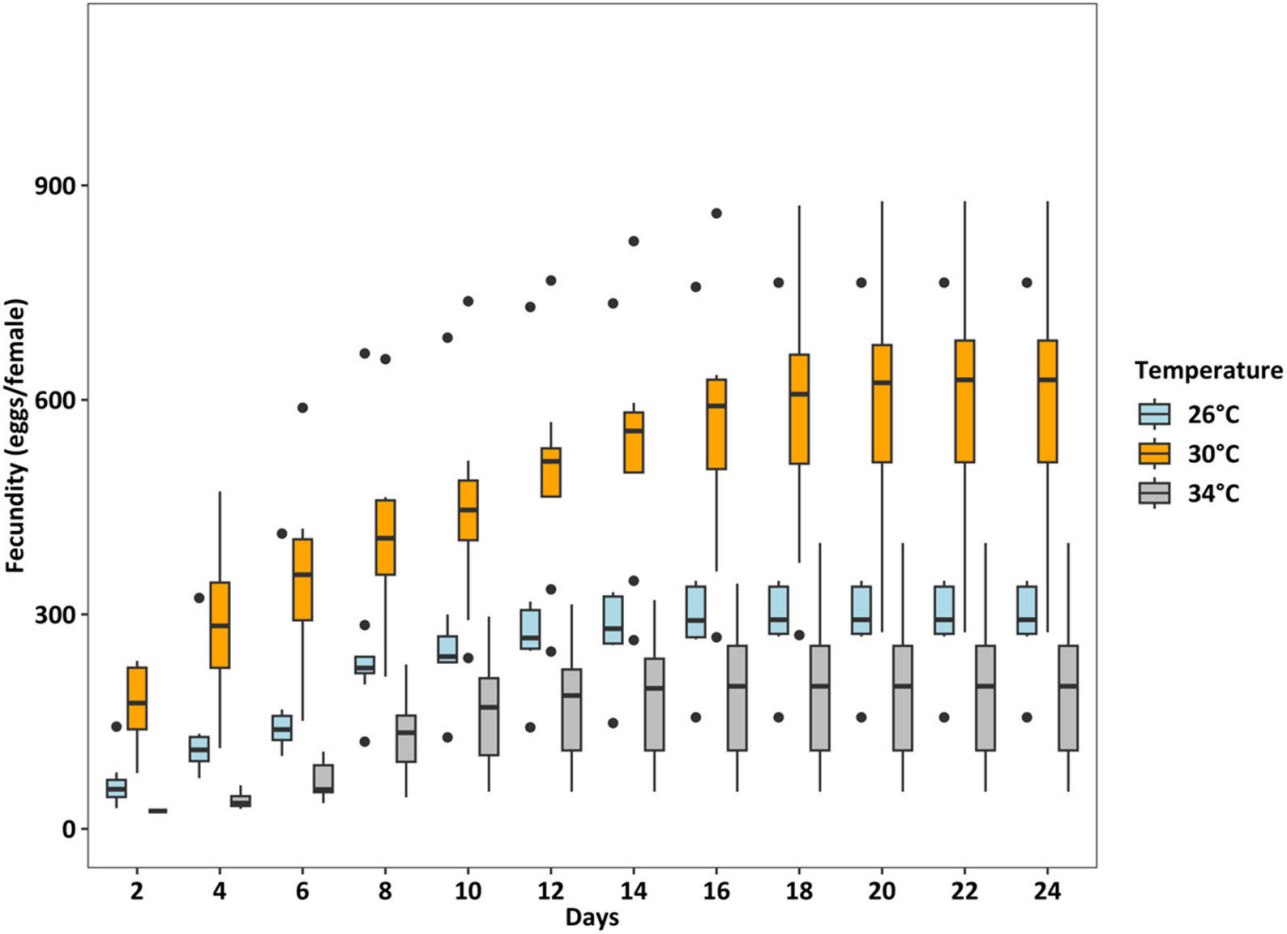
Fecundity of *Acartia* sp. at different temperatures. Fecundity was calculated as the cumulative number of eggs per *Acartia* sp. female over time (Days, x‐axis) at different temperatures using glmPQL model for quasipoisson distribution (*R* ^2^ = 0.87). Each box shows the median (line in the middle of the box) and the 1st and 3rd quantiles (upper tale and lower tale). Pairwise comparisons of cumulative eggs among temperatures during the experimental period show higher fecundity at 30°C than at 26°C and lowest at 34°C (Tukey HSD posthoc test, *p* < 0.05). Details on pairwise comparisons Emmeans are provided in Data S3.

**FIGURE 7:**
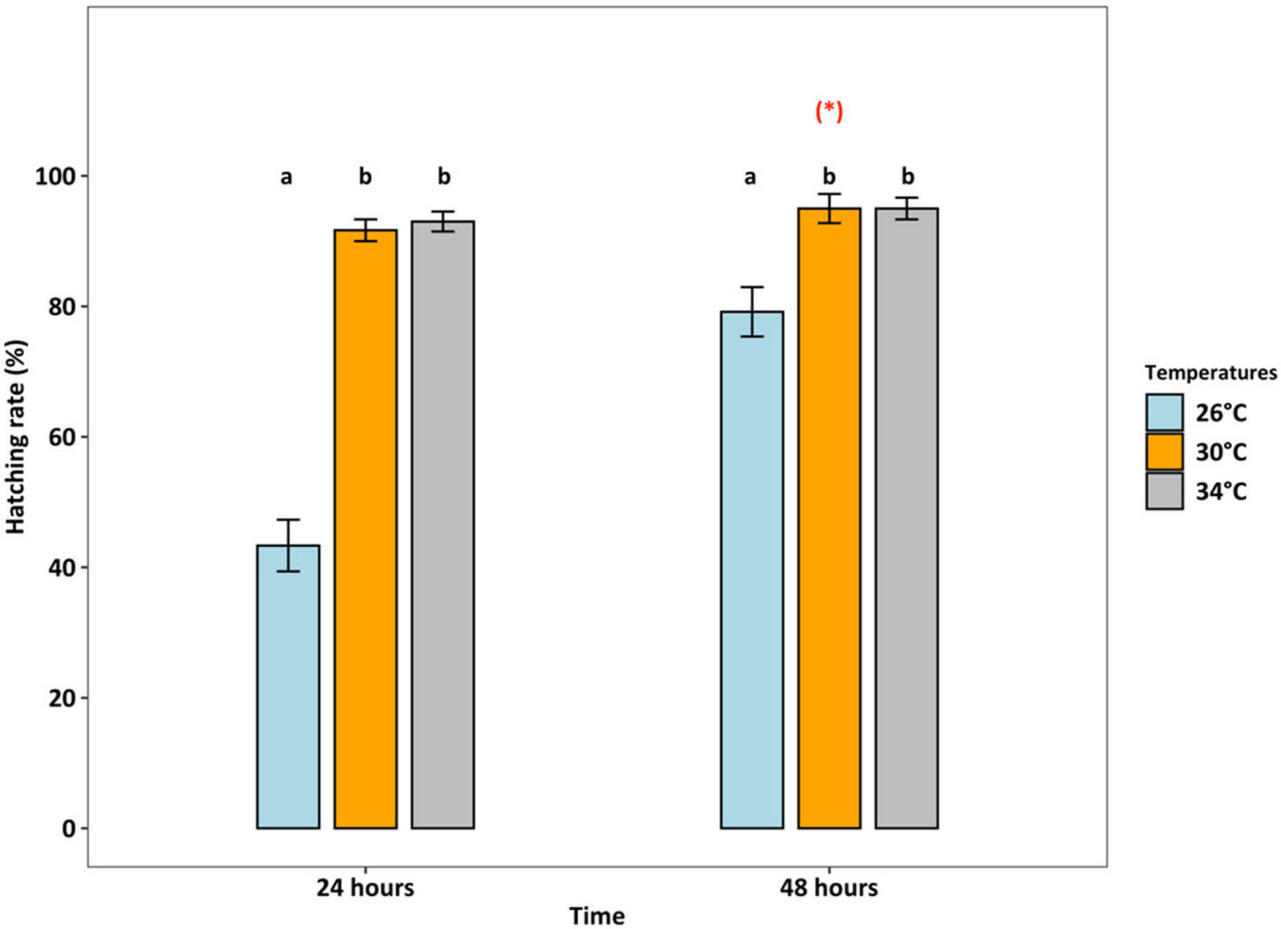
Hatching rate (%, y‐axis) of *Acartia* sp. at different times (24 and 48 h, y‐axis) at three different temperatures (GLM model for quasibinomial distribution, *R* ^2^ = 0.77). Different letters indicate significant differences among temperature treatments at each time point (Tukey HSD post hoc test, *p* < 0.05), and a red asterisk indicates the significant difference between the two‐time points (Tukey HSD post hoc test, *p* < 0.001). Bars and error bars present data as Mean ± SE. Details on pairwise comparisons Emmeans are provided in Data S3.

Survival rates were lowest (36.25%) at 34°C (*p* < 0.001), which was two times lower than at 26°C and 30°C (both > 80%) (Figure 4). At 26°C, the female: male ratio was 50%:50%, but at 30°C and 34°C, the percentage of females accounted for only one‐third of adults (temperature effect, *p* < 0.01, Figure 5). Fecundity was the highest at 30°C, intermediated at 26°C, and was lowest at 34°C (*p* < 0.01, Figure 6). Hatching time was shorter at 30°C and 34°C than at 26°C. At 30°C and 34°C, most eggs hatched within 24 h, which was two times higher than the hatching rate at 26°C (*p* < 0.001, Figure 7). After 48 h of incubation, the hatching success at 30°C and 34°C leveled off (> 90%), while at 26°C it doubled and reached 80% (*p* < 0.001, Figure 7).

### 3.2 16S rRNA Amplicon Sequencing

**FIGURE 8:**
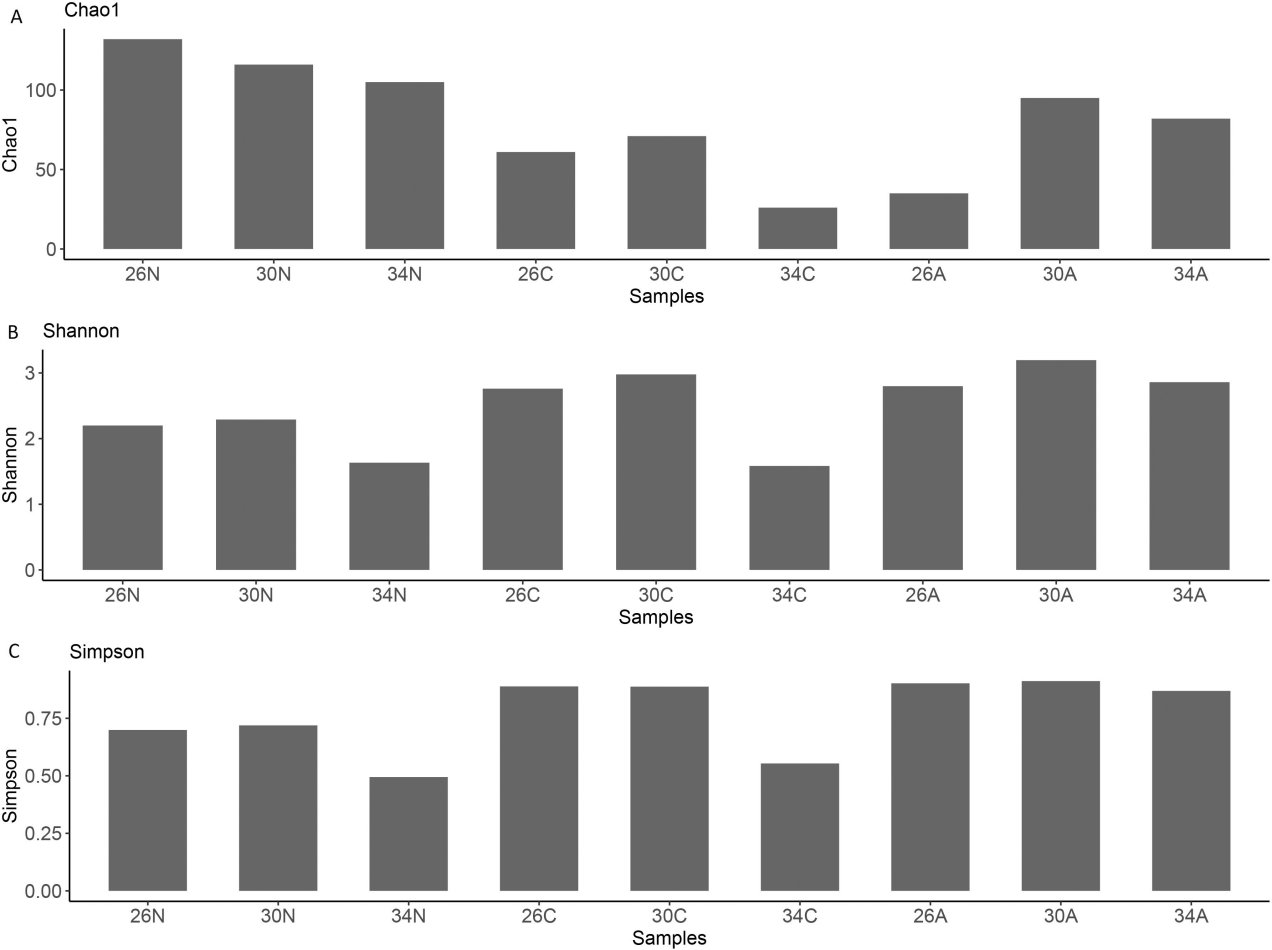
Alpha diversity indices Chao 1 (A), Shannon (B), and Simpson (C) derived from the QIIME (http://qiime.org/) command *α* rarefaction.

We recorded 1,262,987 reads from nine pooled samples, which corresponded to a total of 392 OTUs (Data S4). The number of OTUs highly varied from 30 to 162 OTUs between different treatments and was not significantly different between temperatures (PERMANOVA, *p* = 0.761). In general, nauplii had a different 16S fingerprint than copepodites and adults (see Figure S2). On average of all temperatures, nauplii had a higher OTU number than copepodites and adults (PERMANOVA, *p* = 0.044). On average of all developmental stages, the temperature had no significant effects on alpha diversity indices (Chao1 *p* = 0.764, Shannon *p* > 0.99, Simpson *p* > 0.99, Figure 8). On average of all temperatures, Chao1 diversity was higher in nauplii than in copepodites and adults (PERMANOVA, *p* = 0.048, Figure 8), while Shannon and Simpson diversity did not differ among stages (PERMANOVAs, Shannon *p* = 0.17, Simpson *p* > 0.99, Figure 8). Due to high variations in the internal microbiomes among temperatures × developmental stages, we will describe the relative abundance of the internal microbiomes of *Acartia* sp. at phylum and genus levels in more detail. Due to the limited replications (one sample per temperature and developmental stage), the below descriptive results of the relative abundance of internal microbiomes in copepod samples should be interpreted with caution.

**FIGURE 9:**
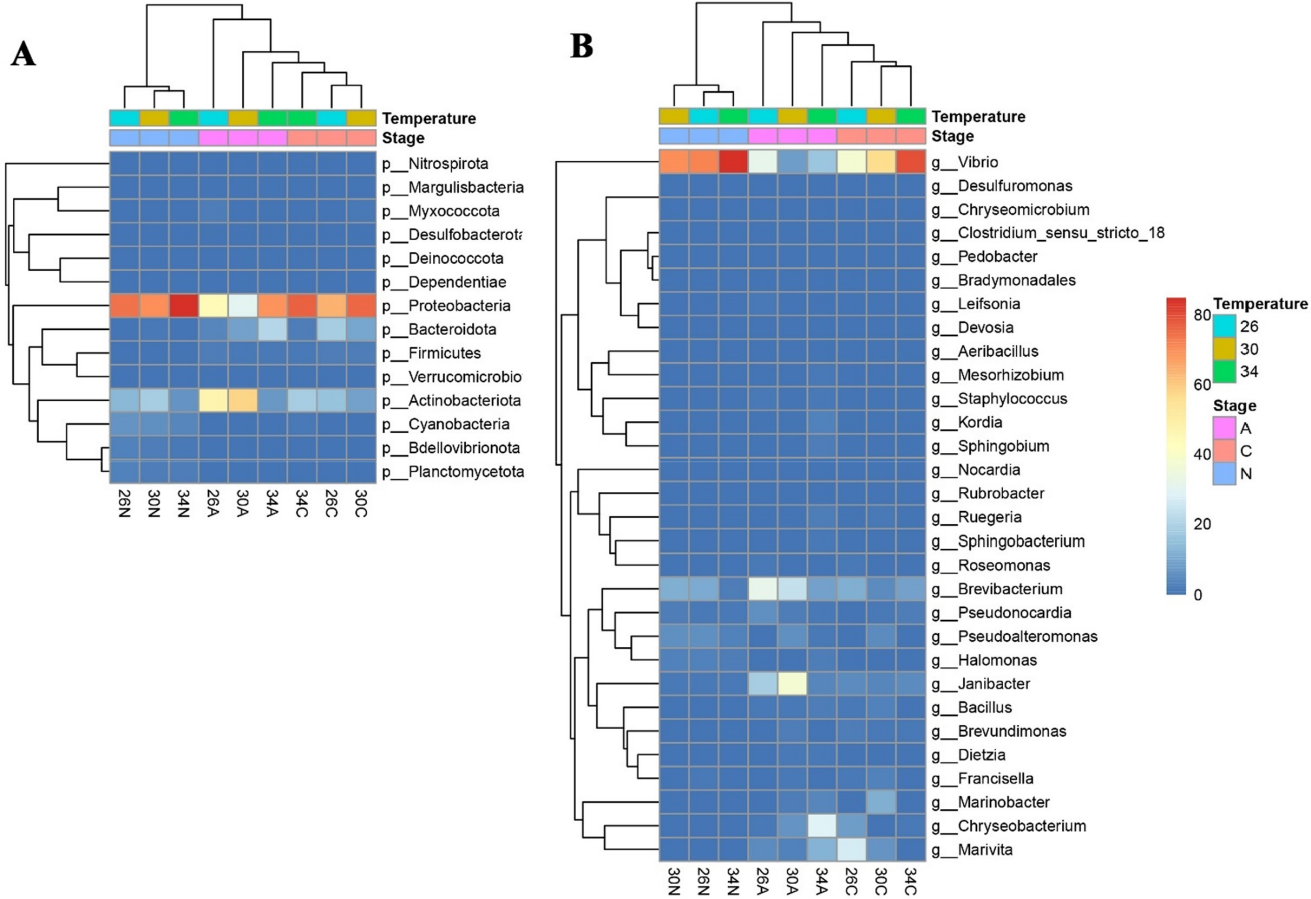
Heatmap revealing the top 14 most abundant phyla (%) and 30 genera (%) of internal microbiomes of the tropical copepod *Acartia* sp. (A) Phylum, and (B) Genus. In the X‐axis: 26N, 30N, and 34N = nauplii samples at 26°C, 30°C, and 34°C; 26C, 30C, and 34C = copepodite samples at 26°C, 30°C, and 34°C; and 26A, 30A, and 34A = adult samples at 26°C, 30°C, and 34°C.

**FIGURE 10:**
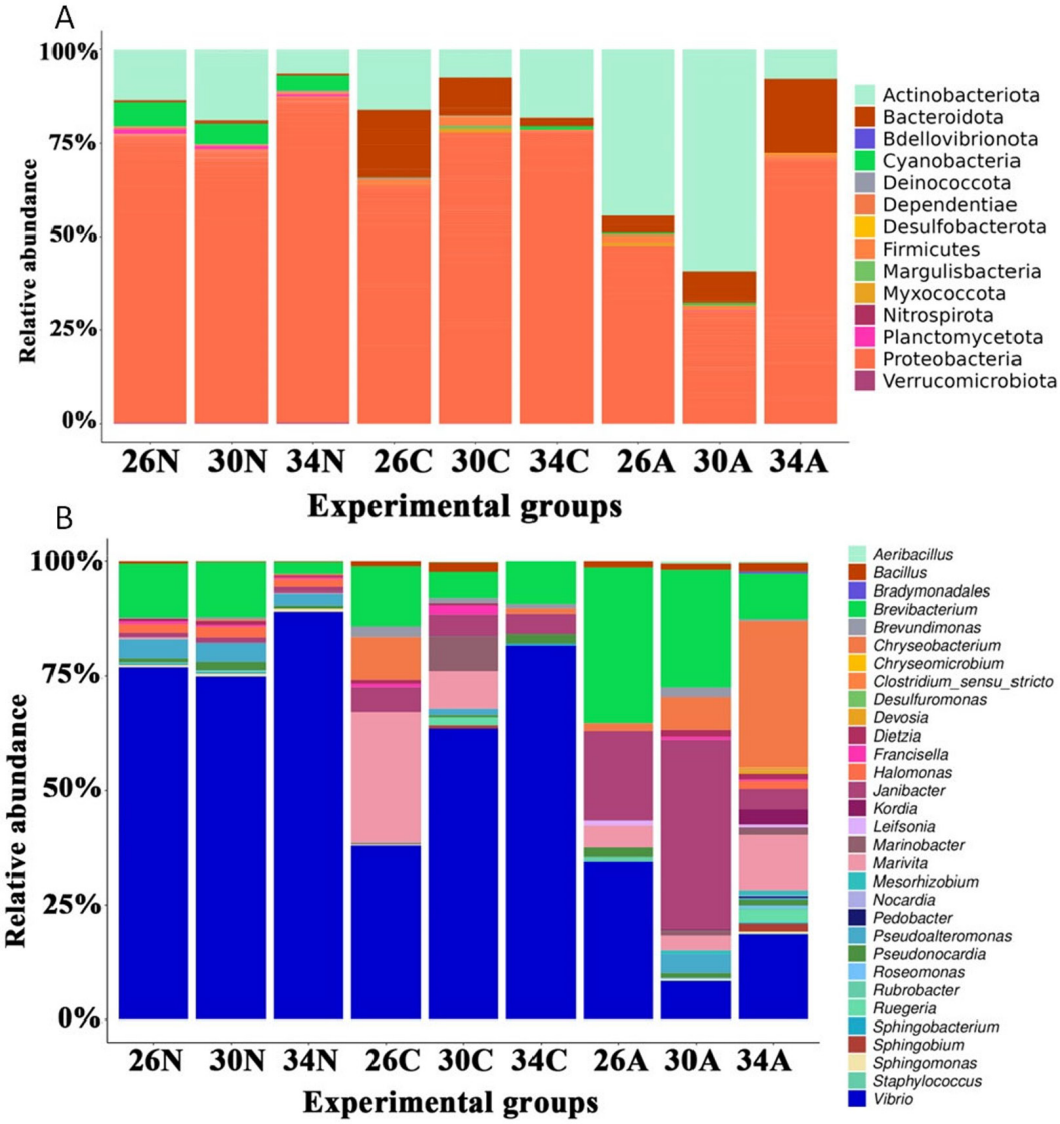
The stacked barplot plots show the microbial composition in samples at classification levels: (A) Phylum and (B) Genus. In the X‐axis: 26N, 30N, and 34N = nauplii samples at 26°C, 30°C and 34°C; 26C, 30C, and 34C = copepodite samples at 26°C, 30°C, and 34°C; and 26A, 30A, and 34A = adult samples at 26°C, 30°C, and 34°C.

At the phylum level (Figures 9A and 10A), Proteobacteria was generally the most abundant group (29.9%–87.2%), except for adult copepods at 30°C. Proteobacteria abundance was higher in nauplii and copepodites than in adults and was higher at 34°C than at 26°C and 30°C. Actinobacteriota is the second most abundant group (6.3%–59.3%) in most samples (Figures 9A and 10A). In adult copepods, Actinobacteriota was more abundant than Proteobacteria at 30°C, while a reverse pattern was observed at 34°C (Figures 9A and 10A). At 26°C, the abundance of these two groups was similar. Bacteroidota was, on average, the third most dominant phylum, but only in copepodites and adults. In copepodites, Bacteroidota abundance was highest at 26°C (18.2%), and their abundance was reduced at higher temperatures (Figures 9A and 10A). In the adults, Bacteroidota abundance was increased at higher temperatures, reaching 20% at 34°C (Figures 9A and 10A). Other phyla, such as Bdellovibrionota, Deinococcota, Desulfobacterora, and Planctomycetota, showed a relatively low proportion (< 5%) in the internal microbiome (Figures 9A and 10A).

The five most abundantly identified genera were *Vibrio*, *Janibacter*, *Brevibacterium*, *Chryseobacterium*, and *Marivita* (Figures 9B and 10B). The genus *Vibrio* was dominant in all samples (Figures 9B and 10B), and more common in nauplii and copepodites (37.9%–89.1%) than in the adults (18.7%–34.4%). In nauplii, *Vibrio* was a predominant genus, particularly at 34°C. In copepodite stages, *Vibrio* was also the most abundant genus and showed increased proportion at higher temperatures: 37.9%, 63.4%, and 81.6% at 26°C, 30°C, and 34°C, respectively. In adults, the abundance of *Vibrio* was highest at 26°C and lowest at 30°C. *Marivita* showed an extremely low abundance in nauplii and copepodites at 34°C, while they showed a relatively high abundance in copepodites at 26°C and in adults at 34°C. *Janibacter* abundance was extremely low in nauplii, while their relative abundance increased from copepodites to adults. In general, *Janibacter* showed the highest abundance at 30°C and the lowest abundance at 34°C and this trend was more pronounced in adults than copepodites. *Brevibacterium* was generally more abundant in adults than in copepodites and nauplii and generally showed smaller proportions at higher temperatures (Figures 9B and 10B). In nauplii, *Brevibacterium* remained at ~12% at 26°C and 30°C, but its relative abundance decreased approximately five times at 34°C (2.6%). A similar trend was observed for the adult copepods. One exception was that the *Brevibacterium* abundance was slightly higher in copepodites at 34°C compared to 30°C. *Chryseobacterium* showed a low abundance in nauplii and copepodites at 30°C and 34°C. However, it was the fourth most abundant genus in copepodites at 26°C. In adults, the relative abundance of *Chryseobacterium* increased 18 times at 34°C compared to at 26°C, from a relatively low abundance (1.66%) at 26°C to becoming the most abundant genus (29.2%) at 34°C (Figures 9B and 10B). The fifth abundance genus was *Marivita* but their abundance was very low in nauplii. In copepodites, *Marivita* abundance was reduced at higher temperatures, while in adults, they showed an increased abundance at 34°C (Figures 9B and 10B).

## 4 Discussion

In this study, we found strong negative effects of 34°C on the fitness of the tropical *Acartia* sp. Interestingly, warming did not affect the total OTU numbers or alpha biodiversity indices, but the relative abundance of thermophilic and opportunistic Proteobacteria and Bacteroidota increased while Actinobacteriota decreased at 34°C. In the following paragraphs, we will discuss the primary physiological mechanisms underlying warming‐induced reductions in the life history traits and subsequently changes in the internal microbiomes.

### 4.1 Survival, Growth, and Development

Overall, copepod performance improved when the temperature increased from 26°C to 30°C. Indeed, *Acartia* sp. developed faster, had a larger body size, a higher fecundity, and a faster hatching rate at 30°C than at 26°C. The faster development of *Acartia* sp. at 30°C can be explained by a higher feeding rate, which has been observed in other tropical calanoid copepod species (Doan et al. 2018; Nguyen et al. 2020). Surprisingly, most of the studies have found that the size of copepods is smaller at higher temperatures (Doan et al. 2019; Dinh, Cuevas‐Sanchez, et al. 2020; Dinh et al. 2021). A Warming‐induced smaller size is nearly a universal pattern across aquatic invertebrates (Daufresne, Lengfellner, and Sommer 2009) and fish (Cheung, Watson, and Pauly 2013) and has been explained by a faster development with respect to growth rate under elevated temperature (Horne et al. 2019). Yet, this was unlikely the case for *Acartia* sp. as they showed a larger body size of C4, C5, and adults at 30°C than at 26°C. This suggests that *Acartia* sp. could keep a higher growth rate in late copepodite stages with respect to development under this temperature range. Our result is not unique, as a similar pattern has been observed in *Acartia* sp. raised from 8°C to 24°C (Leandro, Tiselius, and Queiroga 2006). As a result, the copepod biomass was also substantially higher at 30°C than at 26°C, particularly for adults.

While increasing the temperature from 30°C to 34°C did not seem to affect the copepod development as indicated by an insignificant difference in the development time between these two temperatures, it reduced survival and adult size. A reduced survival at 34°C suggests that this temperature was lethal to the most sensitive individuals in the populations, thereby removing the most sensitive genotypes. The copepod mortality can be explained by two non‐exclusive mechanisms. First, the respiratory system may fail to provide enough oxygen for cellular metabolisms under extreme temperatures, as explained by the Oxygen and Capacity Limited Thermal Tolerance hypothesis (OCLTT, Pörtner, Bock, and Mark 2017). Second, extreme temperatures may impair ATP (Harada, Healy, and Burton 2019) and protein synthesis (Barreto, Schoville, and Burton 2015). A reduced adult size may be a tradeoff between thermal tolerance and growth rate; the fastest‐growing copepods with larger body sizes may also be the most thermal‐sensitive ones (Sasaki et al. 2019). Importantly, the number of days that the coastal water temperatures go beyond 34°C is increasing in the South of Vietnam (Dinh et al. in prep.), especially under MHWs (Feng et al. 2022; Frölicher, Fischer, and Gruber 2018), which raises a serious concern about the abundance of this species, and also other tropical copepod species (Ibarbalz et al. 2019).

### 4.2 Sex Ratio and Reproduction

While the percentage of females and males was the same at 26°C, males were much more abundant at 30°C and 34°C. The imbalance of females and males in the population under elevated temperatures may have implications for population growth and dynamics. Indeed, females play an important role in maintaining the high productivity of the population, having larger biomass and longer adult lifespan (Truong et al. 2020; Kiørboe 2006). The fecundity increased when increasing temperature from 26°C to 30°C, but at 34°C it was strongly reduced, even lower than the fecundity at 26°C. The extreme temperature‐induced reduction in fecundity could be associated with an increase in energy expenditure for coping with thermal stress. A previous study has shown that the oxygen consumption of a tropical calanoid copepod *P*. *annandale*i increased rapidly at temperatures above 32°C (Lehette et al. 2016). The energetic constraints may be further enhanced by reduced feeding, thereby lowering energy intake under 34°C as a similar pattern has been observed in *P*. *annandalei* at the same study location (Doan et al. 2018). A reduced fecundity together with a reduced proportion of females as indicated by a lower female: male ratio may contribute to decreased population growth at 34°C. Future studies should investigate the mechanisms contributing to a lower reproduction rate. For example, it would be interesting to examine if extreme temperatures could reduce sperm and egg quality, thereby lowering the hatching success.

### 4.3 Microbiomes

Increasing temperature did not change the number of OTUs in the copepods, partly because of a high variation in the number of OTUs within groups in each treatment. This result was in contrast with previous findings of warming‐reduced internal microbiome diversity in other ectotherms (Bestion et al. 2017; Li et al. 2018). Therefore, besides the changes in the physiology of copepods, the reduced performance of *Acartia* sp. at 34°C may not link to changes in the OTU numbers, but rather changes in the relative abundance of different groups of internal bacteria (more details below). Internal microbiomes can affect the survival of the host by several pathways. Under heat stress, the microbiome can stimulate the host gene expression of various stress response pathways, including the upregulation of genes encoding heat shock proteins, antioxidant resistance, and cytoskeleton (reviewed in Fontaine and Kohl 2023). Microbial groups can also produce diverse metabolites and proteins that may help the host cope with thermal stress (Burke, Fiehn, and Moran 2010). Warming‐induced reduction in bacteria diversity, especially beneficial groups, potentially negatively affects survival (Li et al. 2018). The negative effect of warming on survival could be intensified by an increased abundance of opportunistic pathogenic bacteria such as species in the genera Vibrio and *Chryseobacterium* (see, e.g., in the mussel *Mytilus galloprovincialis*, Li et al. 2018, 2019).

Proteobacteria was the most dominant phylum in specimens of *Acartia* sp., similar to other marine invertebrates such as marine shrimps (Huang et al. 2016; Rungrassamee et al. 2013), the Mediterranean mussels, *M*. *galloprovincialis* (Li et al. 2019), or the tropical high‐supratidal oyster, *Isognomon nucleus* (Arromrak et al. 2024). Overall, Proteobacteria abundance was generally decreased from nauplii to copepodites, particularly in the adults. The abundance of Proteobacteria in nauplii and copepodites was likely associated with the abundance of the genus *Vibrio*. In the adults, while Proteobacteria was still predominant at 34°C, this did not match with the abundance of the genus *Vibrio*, suggesting that other genera of this phylum may increase their abundance in the adults. Therefore, determining those genera would be of crucial importance to assess the comprehensive role of Proteobacteria in shaping the capacity of *Acartia* sp. to extreme temperatures.

The temperature effects on the relative abundance of Proteobacteria seemed stage‐specific, but showed an overall higher abundance at 34°C than in the other two lower temperatures. In the adults, this increased relative abundance of Proteobacteria was particularly pronounced at 34°C, but it was mostly because of a lower relative abundance of this group at two lower temperatures than in nauplii and copepodites. A previous study on a cold‐adapted copepod (*T*. *kingsejongensis*) also showed an increase in the relative abundance of Proteobacteria at a temperature range of 2°C–15°C (Oh et al. 2022), but no comparable studies have been done for copepods exposing them to extremely high temperatures as in our study. The general increase in the relative abundance of Proteobacteria in *Acartia* sp. at 34°C seems to reflect a high thermal adaptation of this bacterial group (Grzymski et al. 2008).

Proteobacteria are involved in various vital processes in nutrient provisioning, followed by digestion and detoxification (Jing, Qi, and Wang 2020) thus warming‐induced increases in the relative abundance of Proteobacteria in *Acartia* sp. may play an important role in copepod's responses to extreme temperatures during the heatwave periods. The dynamics of the genus *Vibrio* may be an important factor shaping the responses of nauplii and copepodites to the temperature. The genus *Vibrio* has been known to contain numerous genes, including those genes encoding chitinase and virulence factors (Almada and Tarrant 2016; Suginta et al. 2000). The increased relative abundance of *Vibrio* bacteria at higher temperatures in nauplii and copepodites may increase the chitinase activity, thus, it could be associated with faster *Acartia* sp. growth. This can be partly associated with the shorter prosomal length of *Acartia* sp. from the copepodite C4 to adults at 34°C and 30°C. The virulence factor of *Vibrio* bacteria also increases at higher temperatures, which could increase the incidence of disease‐caused mortality in copepods (Roux et al. 2015). In other studies, *Vibrio* bacteria increase their abundance under warmer temperatures, causing diseases in corals and could be an additional mechanism triggering mass mortality events in the coastal Mediterranean Sea (Vezzulli et al. 2010). MHWs could also promote the proliferation of pathogenic *Vibrio* species, causing sponge diseases and mass mortality (Dinçtürk et al. 2023).

Actinobacteria was overall the second most dominant bacterial group in *Acartia* sp. In nauplii and adults, their relative abundance was highest at 30°C and rapidly decreased at 34°C, while copepodites showed the lowest abundance at 30°C. They play various roles in the degradation or decomposition of polysaccharides, chitin, proteins, and lipids and have an antagonistic or competitive effect on pathogenic bacteria (Hazarika and Thakur 2020). The temperature‐induced changes in the relative abundance of Actinobacteria were in the opposite direction from the changes in the Proteobacteria abundance, which were reflected by the dynamic of the genus *Brevibacterium* in the nauplii and the genus *Janibacter* in copepodites and adults at 26°C and 30°C, but not in adults at 34°C. Similar changes in the relative abundance of Proteobacteria and Actinobacteria in response to extreme temperatures have been observed in other marine species, such as sponges (Webster, Cobb, and Negri 2008) and terrestrial isopods (Horváthová et al. 2019). The relative abundance of Actinobacteria has been shown to have a positive correlation with the growth of the host such as isopods, but this correlation was no longer held under warming (Horváthová et al. 2019). Actinobacteria have been known to produce antibiotics that help the host cope with diseases (Horváthová et al. 2019). The genus *Brevibacterium* could degrade various organic chemical compounds in corals, which can help them cope with coastal pollutants (Mahmoud and Kalendar 2016). A reduced abundance of Actinobacteria at 34°C might hinder copepod growth and development and reduce survival.

Bacteroidota was nearly absent in the nauplii, while this phylum is generally one of the abundant phyla in crustacean nauplii such as *Artermia* (Xu et al. 2023), including copepods (Amador‐Marrero et al. 2024). The changes in the relative abundance of Bacteroidota in copepodites and adults were in two opposite directions; warming reduced Bacteroidota abundance in copepodites while increasing it in the adults. The changes in the Bacteroidota were likely related to changes in the abundance of the genus *Chryseobacterium*. Species in the phylum Bacteroidota have been known to play important roles in carbohydrate catabolism, as well as in amino acid and protein utilization in humans (Flint et al. 2012). In marine invertebrates, their abundance of Bacteroidota is suggested to play a role in pyruvate metabolism, glyoxylate, and dicarboxylate metabolism (Liao et al. 2023), which may be especially important for the fast growth of copepodites and maintaining complex biological functions in adults.

Other bacterial groups only accounted for less than 5% of the internal copepod microbiomes and showed inconsistent or invisible trends in their relative abundance in different stages or temperatures. While these groups may play a certain role in copepod digestion, or detoxification, their overall role may be minor. The number of OTUs and Chao1 diversity were higher in nauplii than in copepodites and adults. This was in agreement with previous findings in the only available study on the cold‐adapted Antarctic copepod *T*. *kingsejongensis*, which showed a higher diversity of the fecal microbiomes in nauplii than in adults (Oh et al. 2022). Further investigations are essential to explore the mechanisms for a higher diversity of internal microbiomes in nauplii than in later developmental stages of copepods.

## 5 Conclusions and Future Perspectives

Our results highlight that thermal stress can reduce the fitness of copepods and population growth by reducing survival and fecundity while not changing the total number of OTUs. Rather, the increased relative abundance of Proteobacteria and Bacteroidota, mostly related to the dominance of the genera *Vibrio* and *Chryseobacterium* together with a reduced relative abundance of Actinobacteria reflected in the genera *Brevibacterium* and *Janibacter*, could be associated with the reduced copepod growth, reproduction and survival. Profiling the functional role of all internal bacterial groups in relation to changes in copepod fitness as a function of the temperature will fundamentally advance our mechanistic understanding of the ecological responses of tropical copepods and, more generally, marine invertebrates to a warming climate. The results of this study provide novel knowledge to explain why the internal microbiomes may play a role in the decline in the abundance of tropical copepods under climate change (Grønning et al. 2019; Dinh, Cuevas‐Sanchez, et al. 2020; Dinh, Dinh, et al. 2020; Ibarbalz et al. 2019). These results are complementary to our previous studies that showed multiple lines of evidence that MHWs could reduce the fitness of tropical copepods, within and across multiple generations by impairing copepod physiology (Doan et al. 2019; Dinh, Cuevas‐Sanchez, et al. 2020; Dinh, Dinh, et al. 2020; Dinh et al. 2021; Truong et al. 2020, 2022). From a broader perspective, these results may help to explain why MHWs can cause a substantial decline in the hyperdiversity of tropical marine ecosystems (Barlow et al. 2018; Garrabou et al. 2009, 2019; Smale et al. 2019; Worm et al. 2006).

## Author Contributions

**Quyen D. H. Vu:** conceptualization (lead), data curation (lead), formal analysis (lead), funding acquisition (lead), investigation (lead), methodology (lead), project administration (lead), resources (lead), software (lead), validation (lead), visualization (lead), writing – original draft (lead), writing – review and editing (lead). **Linh P. Pham:** conceptualization (equal), data curation (lead), formal analysis (lead), investigation (equal), methodology (equal), validation (lead), visualization (lead), writing – original draft (lead), writing – review and editing (lead). **Oanh T. Truong:** data curation (equal), formal analysis (equal), investigation (equal), methodology (equal), writing – review and editing (equal). **Sang Q. Tran:** data curation (equal), formal analysis (equal), investigation (equal), methodology (equal), writing – review and editing (equal). **Canh V. Bui:** data curation (equal), investigation (equal), writing – review and editing (equal). **Minh‐Hoang Le:** conceptualization (equal), methodology (equal), writing – original draft (equal), writing – review and editing (equal). **Binh T. Dang:** funding acquisition (equal), investigation (equal), methodology (equal), supervision (equal), writing – review and editing (equal). **Khuong V. Dinh:** conceptualization (lead), supervision (lead), writing – original draft (lead), writing – review and editing (lead).

## Conflicts of Interest

The authors declare no conflicts of interest.

## Supporting information


Data S1.

## Data Availability

All original datasets and codes used in this study are provided in Data S5.
