# Supplementary material for: Extreme Temperatures Reduce Copepod Performance and Change the Relative Abundance of Internal Microbiota
Source: Ecol Evol. 2024 Oct 11;14(10):e70408. doi: 10.1002/ece3.70408 (PMC11470155; doi:10.1002/ece3.70408)
Supplement: Supplementary file 1 — Data S1. [file ECE3-14-e70408-s001.zip › Vu et al. Supplement S1 Rarefraction curves.docx]

**Supplement S1. Rarefraction curves**


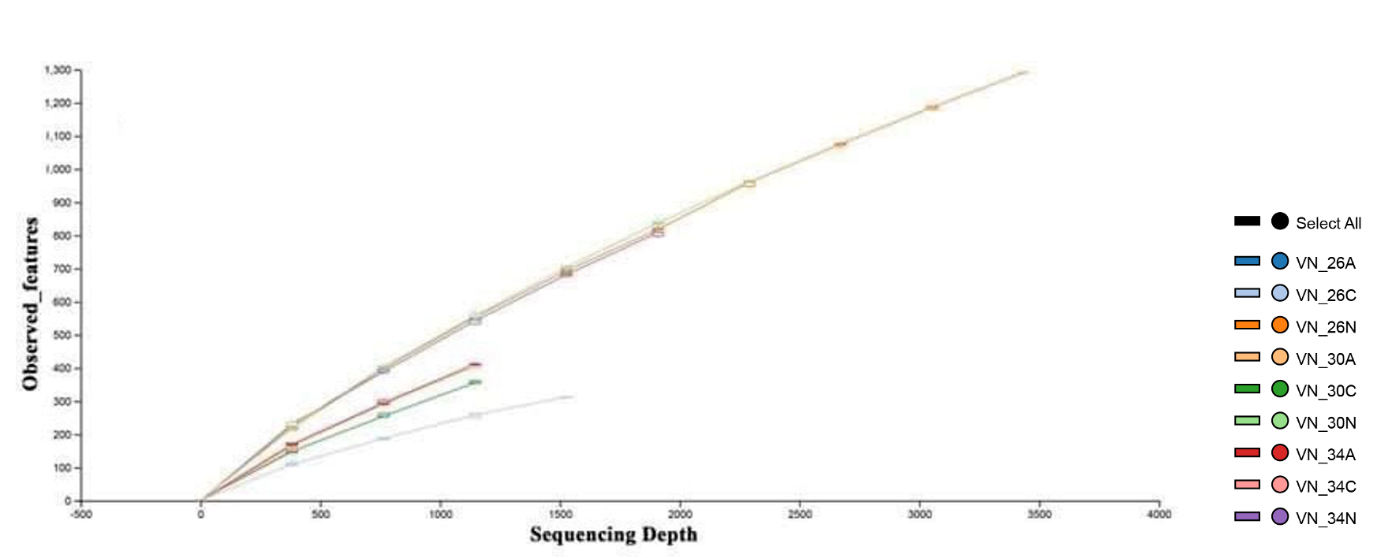


Figure S1. Rarefaction curve for visual comparison of observed OTUs/features at different sequencing depths. From top to bottom: VN_26A = Adults at 26°C, VN_26C = Copepodites at 26°C, VN_26N = Nauplii at 26°C, VN_30A = Adults at 30°C, VN_30C = Copepodites at 30°C, VN_30N = Nauplii at 30°C, VN_34A = Adults at 34°C, VN_ 34C = Copepodites at 34°C, VN_34N = Nauplii at 34°C
