# Supplementary material for: Extreme Temperatures Reduce Copepod Performance and Change the Relative Abundance of Internal Microbiota
Source: Ecol Evol. 2024 Oct 11;14(10):e70408. doi: 10.1002/ece3.70408 (PMC11470155; doi:10.1002/ece3.70408)

### **Appendix S2:** Community composition

We perform principal coordinate analyses (PcoA) based on comparing the Bray–Curtis dissimilarity distance and Jaccard distance.

The Bray–Curtis dissimilarity distance, a key metric in our analysis, was determined by comparing two samples. This comparison is based on the presence rate (percentage) or the number of species co-present sequences between the samples. In other words, it measures the dissimilarity between two samples based on the species they share.

Another important metric in our analysis was the Jaccard distance, which is determined by comparing two samples. This comparison is based on the presence or absence of a species between the two samples, regardless of that species' distribution rate. In simpler terms, it measures the dissimilarity between two samples based on the species they do not share.

(A)


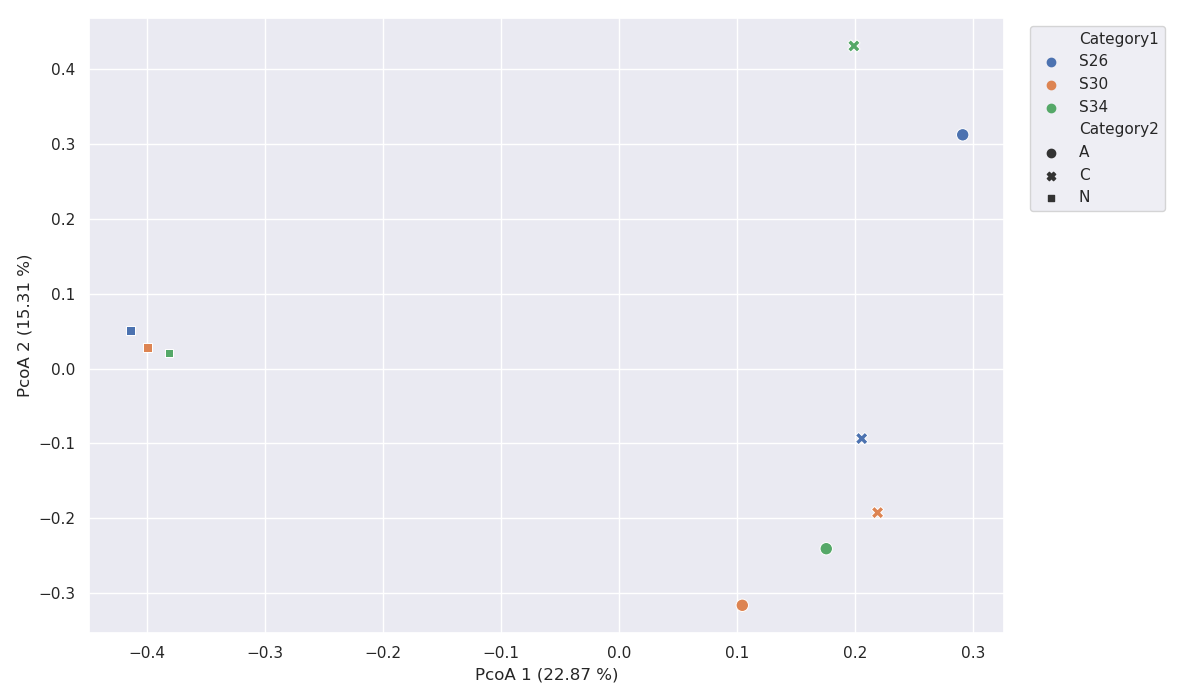


(B)


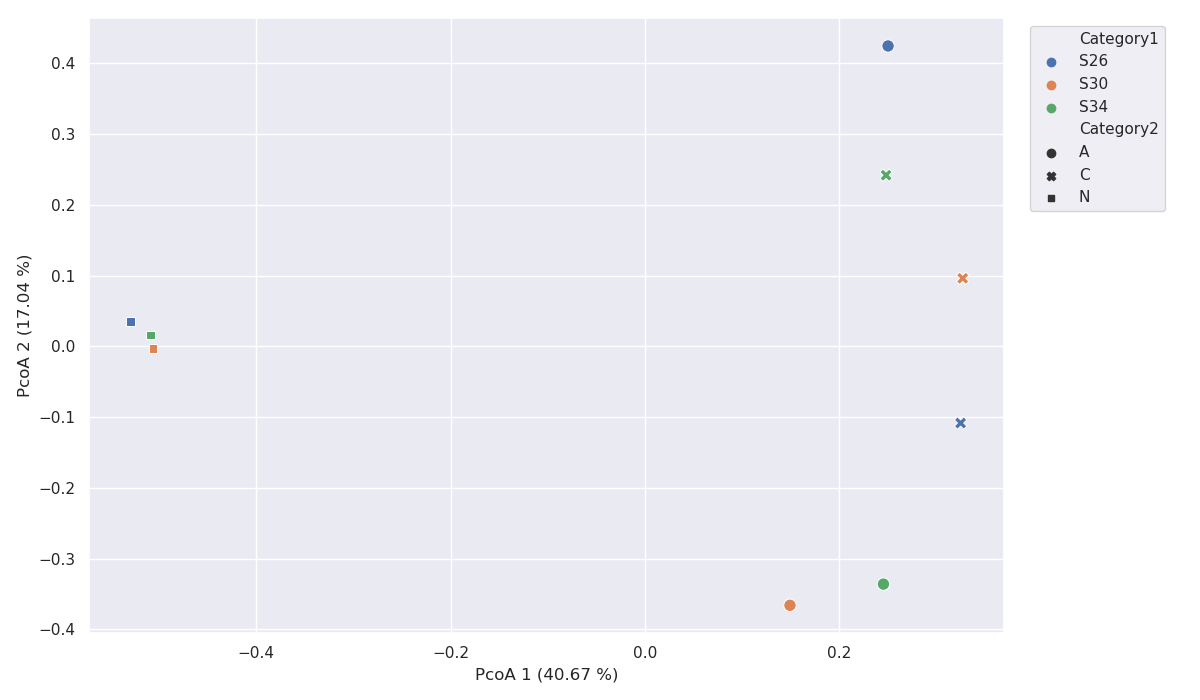


Figure S2 shows the PCoA analysis of groups of samples, with each dot on the graph representing the characteristics of a sample's entire microbiome. Each dot's shape, size, or color represents groups of sample metadata information, helping to distinguish it well on the graph. The percentage value shown on each axis (PCoA1 and PcoA2) represents the percentage of variation that that axis can explain. Samples with similar characteristics will be closer to each other than samples with different characteristics, forming clusters on the graph. (A) based on Bray–Curtis dissimilarity. (B) based on Jaccard distance.

To determine if there is a statistically significant difference between metadata groups, we perform a test PERMANOVA (Table S1). None of the groups showed significant differences.

Table S1. Permanova test results compare the beta index between sample groups. (A) based on Bray–Curtis dissimilarity. (B) based on Jaccard distance.


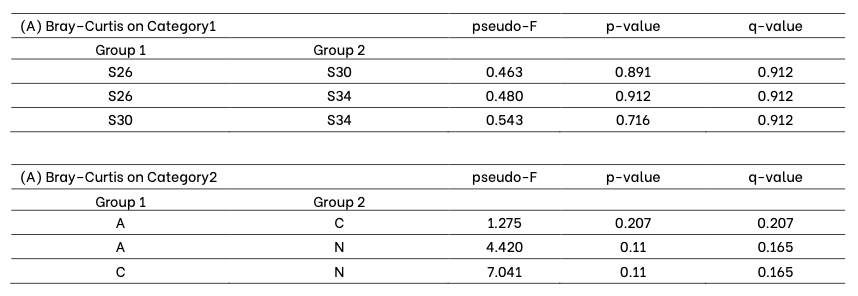


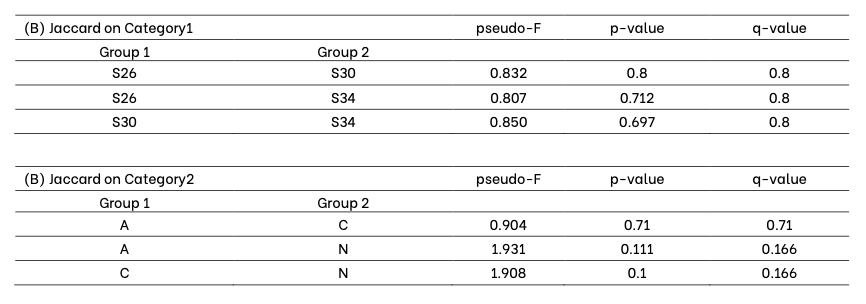

Supplement: Supplementary file 1 — Data S1. [file ECE3-14-e70408-s001.zip › Vu et al. Supplement S2 Principal coordinate analysis.docx]
